# A Mobile Videoconference-Based Intervention on Stress Reduction and Resilience Enhancement in Employees: Randomized Controlled Trial

**DOI:** 10.2196/10760

**Published:** 2018-10-22

**Authors:** Johanna Inyang Kim, Je-Yeon Yun, Heyeon Park, Suk-Young Park, Youngsheen Ahn, Hansol Lee, Tae-Kwon Kim, Sooran Yoon, Young-Joon Lee, Sohee Oh, John W Denninger, Bung-Nyun Kim, Jeong-Hyun Kim

**Affiliations:** 1 Hanyang University Medical Center Seoul Republic Of Korea; 2 Seoul National University Hospital Seoul Republic Of Korea; 3 Seoul National University College of Medicine Seoul Republic Of Korea; 4 Seoul National University Bundang Hospital Seongnam Republic Of Korea; 5 Seoultop Psychiatric Clinic Seoul Republic Of Korea; 6 Korean Academy of Gifted Education Seoul Republic Of Korea; 7 Aimmed Company Ltd Seoul Republic Of Korea; 8 Seoul Metropolitan Government Seoul National University Boramae Medical Center Seoul Republic Of Korea; 9 Benson-Henry Institue for Mind Body Medicine, Massachusetts General Hospital Boston, MA United States; 10 Department of Psychiatry, Harvard Medical School Boston, MA United States; 11 Human Behavioral Medical Institute Seoul National University Medical Research Center Seoul Republic Of Korea

**Keywords:** employees, mobile phone, randomized controlled trial, resilience enhancement, stress reduction, videoconferencing

## Abstract

**Background:**

Videoconferencing-based treatments have shown great potential in increasing engagement and compliance by decreasing the barriers of time and distance. In general, employees tend to experience a lot of stress, but find it difficult to visit a clinic during office hours.

**Objective:**

The purpose of this study was to investigate the effectiveness of a mobile videoconference-based intervention for stress reduction and resilience enhancement in employees.

**Methods:**

In total, 81 participants were randomly allocated to one of the three conditions: mobile videoconferencing, in-person, and self-care; of these, 72 completed the study. All participants underwent assessment via self-reported questionnaires before, immediately after, and 1 month after the intervention. Intervention lasted for 4 weeks and consisted of elements of cognitive behavioral therapy, positive psychology, and meditation. Changes in clinical variables regarding stress and resilience across time were compared between treatment conditions.

**Results:**

There were significant condition × time effects on variables measuring perceived stress, resilience, emotional labor, and sleep, demonstrating significantly differential effects across time according to treatment condition. Moreover, there were significant effects of condition on perceived stress and occupational stress. There were no significant differences in any variable between the mobile videoconferencing and in-person conditions at 1 month after the intervention.

**Conclusions:**

Results indicate that both mobile videoconferencing and in-person interventions were comparably effective in decreasing stress and enhancing resilience. Further studies with a larger sample size and a longer follow-up period are warranted to investigate the long-term effect of mobile videoconferencing interventions.

**Trial Registration:**

ClinicalTrials.gov identifier NCT03256682; https://clinicaltrials.gov/ct2/show/NCT03256682 (Archived by WebCite at http://www.webcitation.org/71W77bwnR)

## Introduction

Stress is a major public health concern. It can cause serious psychological and physical problems such as fatigue, sleeping problems, coronary diseases, depression, and even related mortality [1-4]. Hence, proper stress management for employees is particularly important; work-related stress affects approximately 28% of all workers [5]. Stress has also been reported to be a major factor in up to 80% of all work-related injuries and 40% of workplace turnovers [6]. Data from the American Institute of Stress show that job stress in the United States accounts for over US $300 billion annually as a result of increased absenteeism, employee turnover, diminished productivity, medical, legal, and insurance expenses, and worker compensation [7].

Traditional face-to-face or in-person stress reduction interventions for employees are proven to be effective, with small-to-moderate effect sizes [8]. However, a major barrier to managing stress is limited access due to time and location constraints, as it may be difficult for employees to find the time to see a therapist during work hours on weekdays [9]. The success of nonpsychopharmacological psychiatric treatment is related to treatment adherence, which is linked to barriers like time and distance. A useful way to increase access, and thereby increase treatment adherence, is by incorporating telemedicine-based methods. Employees can easily access videoconferencing-based treatments at their convenience, ie, during break hours in the workplace or after office hours at home [10].

There is a bulk of evidence suggesting that videoconferencing- based telepsychology is no less effective than in-person treatment in a variety of psychiatric disorders [11], including depression [12], panic disorders [13], obsessive compulsive disorder [14], posttraumatic stress disorder [15], and eating disorders [16]. A recent meta-analysis of 26 randomized controlled trials demonstrated noninferiority of remote psychiatric counseling, with respect to both assessment and treatment, compared with in-person counseling [17]. To the best of our knowledge, to date, there has been no study evaluating the effectiveness of videoconferencing in reducing stress and enhancing resilience in the workplace.

An important issue in videoconferencing is equipment. In most previous studies, participants were instructed to use the equipment provided at remote videoconference sites [11]. However, traveling to these sites is no different from traveling to see a counselor; it would still be a barrier to treatment for those whose workplace or home is far from the videoconference site. To further increase accessibility to treatment, we used mobile-based videoconferencing equipment. Access to treatment by smartphones could be particularly beneficial in South Korea, since almost all Korean employees own a smartphone (94% of population), making it one of the countries with the highest smartphone ownership worldwide [18].

The purpose of this study was to compare the effects of mobile videoconference-based intervention on stress reduction and resilience enhancement with that of in-person and self-care methods in Korean employees. We hypothesized that the effectiveness of the mobile videoconference condition would be superior to the control condition (self-care condition), but comparable to the in-person condition.

## Methods

### Participants

Participants were recruited via advertisements at the Seoul National University Hospital and the Seoul National University Bundang Hospital between August 2017 and November 2017 (NCT03256682). The inclusion criteria were (1) age between 19 and 65 years, (2) a score of ≥14 on the Perceived Stress Scale (PSS) at baseline, (3) possession of an Android smartphone, and (4) currently employed full-time. As stress in the workplace is commonly associated with depression, insomnia, and anxiety [1,19] and as stress management interventions have been found to be effective at treating these three conditions [20,21], we included individuals with these conditions as long as the type and dosage of their medication had not changed for the past 6 months. The exclusion criteria were (1) age <19 or >65 years; 2) cognitive disorders, such as intellectual disability or dementia; 3) neurological disorders, including epilepsy, stroke, or others; 4) history of schizophrenia or bipolar I disorder; 5) current report of suicidal ideation; and 6) nonpharmacological treatment or counseling within the past 6 months.

During the screening process, psychiatric diagnoses were confirmed using the Mini-International Neuropsychiatric Interview (MINI), which is a short, structured psychiatric interview designed to detect a wide range of Diagnostic and Statistical Manual of Mental Disorders, Fourth Edition and International Classification of Diseases, Tenth Revision psychiatric disorders [22]. Questions are phrased to allow only “yes” or “no” answers. The Korean version has well-established validity and reliability [23]. MINI was conducted by two psychologists with a master’s degree in education and 2 hours of training in advance.

With a predicted effect size of Cohen *d*=0.4, an alpha level of.05, a desired power of 0.95, and a correlation of 0.5 among repeated measures, the estimated total sample size using G-Power was 69 (23 participants per condition). Considering a drop-out rate of 20%, we aimed to recruit 87 participants.

Written informed consent was obtained from all participants after sufficient explanation of the study. The study protocol was approved by the Institutional Review Board of Seoul National University Bundang Hospital.

### Assessments

Demographic information, including age, gender, length of work (<3 years or ≥3 years), marital status (married and not separated, others), and education status (college education or more, less than a college education), was obtained using a self-reported questionnaire. These demographic variables have been previously found to relate to occupational stress level [24]. The questionnaires were filled on paper. The primary outcome measures of this study were changes in scores of PSS and Brief Resilience Scale (BRS) scales. Changes in scores of scales assessing emotional labor, occupational stress, and insomnia were evaluated as secondary outcomes.

PSS is a 10-item questionnaire used to assess perceived stress [25]. It was designed to measure the degree to which respondents found their life situations unpredictable, uncontrollable, and overbearing. It includes 10 direct queries about incidents that brought upon states of being upset, nervous, stressed, or irritated; four items were worded negatively and the rest positively. Answers were given on a 5-point Likert scale (0: never to 4: very often). Total scores were calculated after reversing the scores from positive items and then summing up all scores. Higher scores indicated higher levels of perceived stress. The Korean version of PSS was found to have a Cronbach alpha=.74 and a test-retest reliability of intraclass correlation coefficient=0.81 [26].

We used the Korean Emotional Labor Scale (KELS) [27] to measure the level of emotional labor. Emotional labor is defined as the process by which workers have to control their feelings in accordance with the organizational demand and occupational role [28,29]. KELS was developed to measure Korean-specific emotional labor and was validated with a nation-wide random sample of 1042 Korean employees. It was based on the literature related to emotional labor [29-35]; emotional labor scales such as the Emotional Labor Inventory [36], Emotional Labor Scale [37], Emotion Work Requirements Scale [38], and Frankfurt Emotion Work Scale [39]; and a focused group interview. KELS has 5 subscales: effort to control emotion (5 items), organizational monitoring system (4 items), demands of emotional labor (3 items), emotional damage (6 items), and organizational support system (7 items). Each item was rated on a 4-point Likert scale, from 1 (not at all) to 4 (very much). Scores for each subscale were calculated based on the scoring method provided by the developers. The possible range for each subscale was 0-100, with higher scores representing higher levels of emotional labor. In this study, we only used the total score.

The level of job stress was measured using the Korean Occupational Stress Scale (KOSS)-Short Form, which is one of the most commonly used questionnaires for assessing job stress in South Korea [40]. It consists of 24 items measured on a 4-point Likert scale (1: never to 4: always). This scale comprises 7 subscales, including job demand, job control, interpersonal conflict, job insecurity, organizational system, lack of reward, and workplace environment. The sum of each subscale was calculated and then converted to 100 points. We used the KOSS total score in the analysis; higher scores indicated a higher level of job stress.

BRS was used to measure individual resilience [41]. It aims to assess the most traditional and original sense of resilience, in other words, “the ability to bounce back from stress [42].” It consists of 6 items measured on a 5-point Likert scale (1: strongly disagree to 5: strongly agree). While other resilience scales measure personal characteristics that may promote positive adaptation, BRS is the only scale that targets and assesses resilience itself.

Insomnia was measured by the Athens Insomnia Scale (AIS), which contains 8 items scored on a 4-point Likert scale [43]. Total AIS scores range from 0 to 24, with higher scores indicating greater symptom severity. AIS has been validated for screening insomnia in South Korean firefighters with good psychometric properties (Cronbach alpha=.88 and item-total correlation=0.73) [44].

All self-reported questionnaires were completed by the participants before treatment, immediately after treatment, and 1 month after treatment.

At posttreatment, participants answered 4 questions about therapeutic alliance. Each item was rated on a 5-point Likert scale (1: disagree strongly to 5: agree strongly). The 4 questions were as follows: (1) “I felt as if the therapist understood me well”; (2) “I felt as if the therapist was paying attention to what I was saying”; (3) “I could tell that the therapist was empathetic by his/her tone of voice”; and (4) “I felt comfortable during therapy sessions.”

### Randomization and Treatment Conditions

We performed 1:1:1 block randomization (stratified by organization) with randomly selected block sizes (3, 6, or 9) using REDCap (Research Electronic Data Capture) tools hosted at the Medical Research Collaborating Center of Seoul National University Bundang Hospital. REDCap generates randomization codes using SAS software. The allocation sequence was concealed to the patients until they had entered the trial and to the investigators until the end of the study. Participants in the mobile videoconference and in-person conditions underwent 50-minute sessions of 1:1 therapy with 1 of 3 psychologists with a master’s degree in education for 4 weeks (one session a week). The protocol of therapy was adapted from the Stress Management and Resilience Training: Relaxation Response Resilience Program (SMART-3RP) [45]. SMART-3RP is an 8-week, 1.5-hour session program developed by the Benson-Henry Institute for Mind Body Medicine at Massachusetts General Hospital. This program is based on the principles of cognitive behavioral therapy and positive psychology in conjunction with methods that elicit a relaxation response. The goals of the program include (1) eliciting a relaxation response through meditation, (2) reducing overall stress reactivity, and (3) increasing connectedness to oneself and others. In this study, we modified the SMART-3RP program into a 4-week program (1-hour per session); a brief summary of each session is presented in Multimedia Appendix 1. The participants in the self-care condition received educational material regarding methods to self-regulate stress and were instructed to read 1 chapter each week for 4 weeks. This material was also provided to the participants in other conditions.

### Apparatus

For mobile videoconferencing we used the “Hello Mindcare” Android app [46], which was developed to provide mobile counseling services. All participants downloaded the app free of charge. Using Web Real-Time Communication, the Hello Mindcare app provides videoconferencing by allowing direct peer-to-peer communication and eliminating the need to install plugins or ActiveX. With a highly secure system, all data shared during videoconference sessions were encoded using Transport Layer Security, 128-bit block encryption algorithm ARIA (Academy, Research Institute, Agency), and Advanced Encryption Standard. The Hello Mindcare app provides a booking system, videoconferencing, document sharing, and workbooks for clients to fill in directly via their smartphones. Therapists used the Hello Mindcare counselor Web to manage schedules, participate in videoconferencing sessions, and check participants’ workbooks.

### Statistical Analysis

The demographic and clinical characteristics of each condition were compared using analysis of variance (ANOVA) for continuous variables and chi-square tests or Fisher exact tests for categorical variables. Condition, time, and condition × time effects on clinical variables were tested using repeated measure ANOVA with age and marital status included as covariates. For post hoc analyses, we conducted pairwise comparisons of the changes in scores at posttreatment (posttreatment score − pretreatment score) and at 1-month follow-up (follow-up score − pretreatment score) by analysis of covariance, with age and marital status included as covariates. All statistical analyses were performed using IBM SPSS Statistics version 22.0 software (IBM Corp, Chicago, IL, USA). A two-tailed *P* value of <.05 was considered statistically significant.

## Results

**Figure 1:**
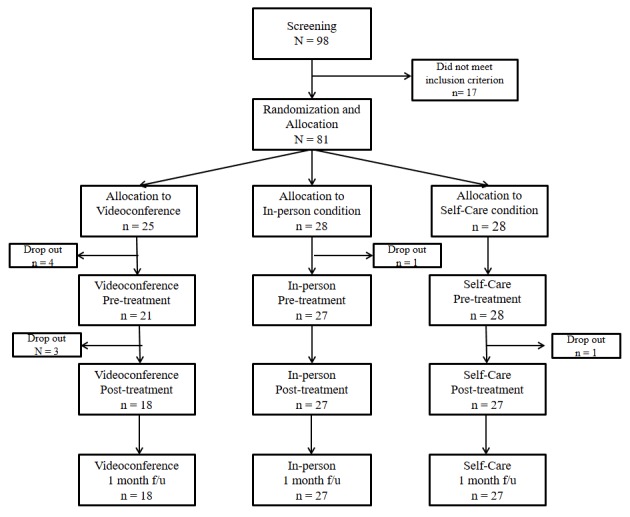
Flowchart of the study process. f/u: follow-up.

Of the 98 individuals screened for this study, 17 did not meet the inclusion criteria; 81 individuals were thus enrolled and randomly allocated to 1 of the 3 conditions. Among them, 4 in the mobile videoconference condition and 1 in the in-person condition dropped out after randomization but before treatment initiation (mobile videoconference condition: 3 had trouble installing the app on their smartphone and 1 refused participation due to difficulty in scheduling appointments; in-person condition: 1 needed psychiatric treatment due to aggravation of psychiatric symptoms). As a result, 21 participants were allocated to the mobile videoconference condition, 27 to the in-person condition, and 28 to the self-care condition; all 81 subjects completed the pretreatment assessment. After the start of treatment, 3 participants in the mobile videoconference condition and 1 participant in the self-care condition dropped out (mobile videoconferencing condition: 2 dropped out due to their personal schedules, 1 complained of unstable Wi-Fi connection; self-care condition: 1 dropped out of because of personal matters, but refused to give a detailed explanation). A total of 18 individuals in the videoconference condition, 27 in the in-person condition, and 27 in the self-care condition completed all 4 sessions of the intervention and underwent the posttreatment and 1-month follow-up assessment (Figure 1). The drop-out rates after treatment engagement were 14% (3/21), 0%, and 3% (1/27) for the mobile videoconferencing, in-person, and self-care conditions, respectively; this distribution was not statistically significant (*P*=.09 by Fisher exact test).

The demographic and clinical characteristics of participants who completed the assessments at all three time points are presented in Table 1. The mean age of participants in the mobile videoconference condition and in-person condition was higher than that of the self-care condition (*P*<.001), and there was a significant difference in marital status among the conditions (*P*=0.03); hence, age and marital status were included as covariates in the main analyses. There were no significant differences in gender, length of work, education status, or baseline scores of the clinical variables. When classifying the occupations of the participants according to the International Standard Classification of Occupations 08, 5 were managers, 26 were professionals, 6 were technicians and associate professionals, 12 were clerical support workers, 19 were service and sales workers, 1 was a plant and machine operator, and 3 had elementary occupations. Overall, 42% (30/72) were hospital employees.

**Figure 2:**
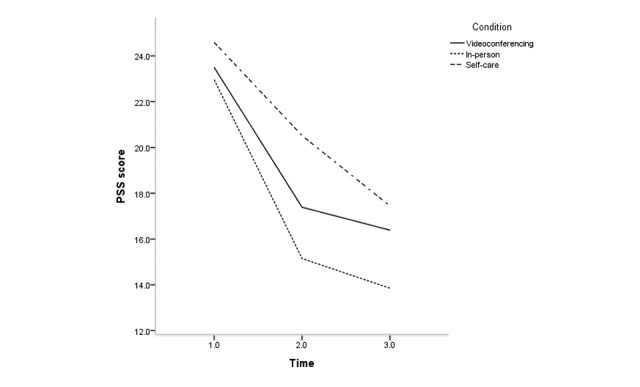
Change in Perceived Stress Scale scores (PESS) across time according to condition.

**Figure 3:**
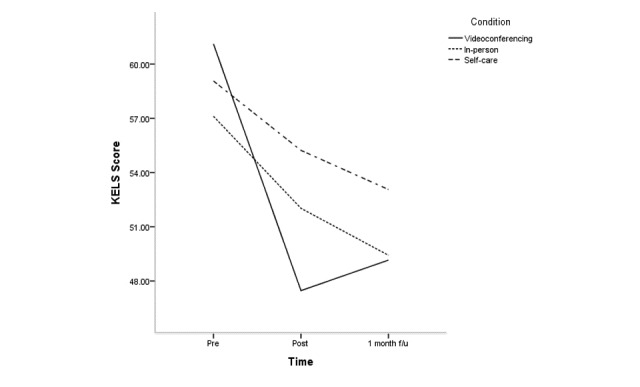
Change in Korean Emotional Labor Scale (KELS) scores across time according to condition.

**Figure 4:**
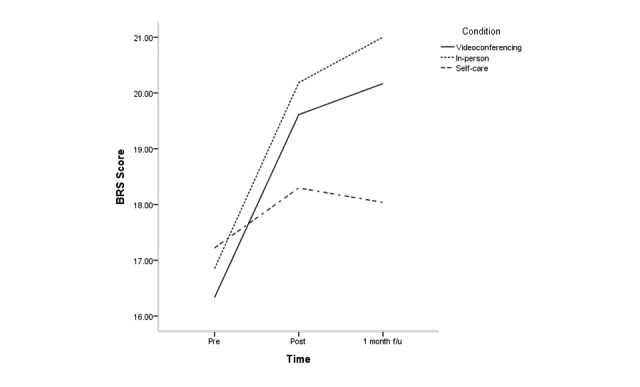
Change in Brief Resilience Scale (BRS) scores across time according to condition.

**Figure 5:**
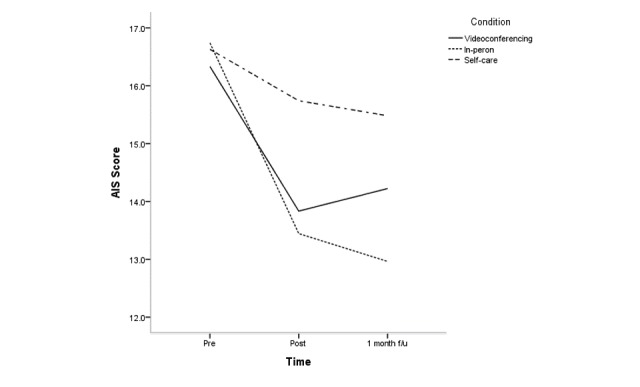
Change in Athens Insomnia Scale (AIS) scores across time according to condition.

The effects of condition, time, and condition × time for all clinical variables are shown in Multimedia Appendix 2; Figures 2-5 depict changes in the PSS, KELS, BRS, and AIS scores across time. The interaction between time and condition was significant for 4 clinical variables (PSS: *F*_3.40_=3.1, *P*=.03; BRS: *F*_3.47_=3.9, *P*=.008; KELS: *F*_3.45_=2.8, *P*=.03; AIS: *F*_3.04_=4.5, *P*=.005). There were significant main effects for condition on PSS (*F*_2_=8.7, *P*<.001) and KOS (*F*_2_=11.6, *P*<.001).

At posttreatment, the mobile videoconferencing condition showed a greater decrease in KELS scores at posttreatment, but this was not significant at follow-up (Multimedia Appendix 2). There were no significant differences in any clinical variable between the mobile videoconferencing condition and in-person condition at follow-up. The mobile videoconferencing condition showed a greater decrease in KELS scores compared with the self-care condition at posttreatment, but this was without significance at follow-up. The mobile videoconferencing condition showed a greater increase in BRS scores at follow-up than the self-care condition. The in-person condition showed a greater decrease in PSS, KOSS, BRS, and AIS scores at posttreatment than the self-care condition, but only KOSS, BRS, and AIS scores were significant at follow-up.

Regarding the 4 questions about therapeutic alliance, there were no differences in the scores between the videoconferencing condition and in-person condition: question 1: average score 4.7 (SD 0.6) versus 4.7 (SD 0.4), *P*=.58; question 2: average score 4.7 (SD 0.6) versus 4.9 (SD 0.3), *P*=.32; question 3: average score 4.6 (SD 0.5) versus 4.9 (SD 0.3), *P*=.06; question 4: average score 4.7 (SD 0.6) vs 4.7 (SD 0.6), *P*=.98.

**Table 1:** Demographic and clinical characteristics of participants in videoconferencing, in-person, and self-care conditions.

Characteristic	Videoconference (n=18)	In-person (n=27)	Self-care (n=27)	*P* value
Age in years, mean (SD)	36.2 (9.2)	36.7 (10.3)	46.6 (9.6)	<.001^a^
Sex (female), n (%)	17 (94.4)	25 (92.6)	25 (92.6)	.97
Education ≥college bachelor degree, n (%)	16 (88.9)	24 (88.9)	18 (66.7)	.07
Marital status (married and unseparated), n (%)	10 (55.6)	17 (63.0)	24 (88.9)	.03
Employed >3 years, n (%)	14 (77.8)	14 (51.9)	19 (70.4)	.16
Perceived Stress Scale, mean (SD)	23.5 (4.2)	23.0 (3.3)	24.6 (4.1)	.30
Brief Resilience Scale, mean (SD)	16.3 (4.1)	16.9 (3.5)	17.2 (4.0)	.75
Korean Emotional Labor Scale, mean (SD)	61.1 (17.1)	57.1 (15.6)	59.1 (14.6)	.70
Korean Occupational Stress Scale, mean (SD)	52.8 (7.7)	53.1 (9.5)	57.5 (9.3)	.12
Athens Insomnia Scale, mean (SD)	16.3 (3.2)	16.7 (4.2)	16.6 (4.1)	.94

^a^Post hoc test by least significant difference; age was less in videoconference and in-person conditions than in self-care conditions.

## Discussion

To the best of our knowledge, this is the first study investigating the effectiveness of a mobile videoconferencing-based intervention for stress reduction and resilience enhancement in employees. Videoconferencing was delivered using a smartphone app, thus combining the merits of both videoconferencing and mobile devices. Overall, there were significantly differential effects across time according to treatment condition on perceived stress, resilience, emotional labor, and sleep. Moreover, there were no significant differences between the mobile videoconferencing and in-person conditions at follow-up, suggesting that the benefits of mobile videoconferencing therapy were comparable to those of in-person therapy.

At follow-up, both the mobile videoconferencing and in-person conditions had significantly enhanced resilience compared with the self-care condition, which was the primary goal of the intervention. Resilience enhancement has been recognized as an important part of stress reduction. Resilience refers to the process that allows individuals to adapt positively despite stress or trauma [47]. Resilience-based interventions emphasize strengths within individuals and community members to persevere and recover from environmental, physical, or emotional stress [48]. Previous research regarding resilience suggests approaches that build on strengths rather than problem-focused strategies [47]. Southwick and Charney suggested 5 essential components to resilience interventions: (1) emotional regulation training to recognize and manage reactivity and impulsivity; (2) cognitive behavioral approaches to reframe thought processes and increase positive emotion; (3) physical health information on exercise, nutrition, sleep, and relaxation to increase protective behavior; (4) social support to build connections with family, peers, and mentors to increase protective factors; and (5) neurobiological components, such as mindfulness-based stress reduction, to increase the ability to manage stress [49]. Our intervention contained most of these components, leading to successful resilience enhancement.

Most studies on videoconferencing-based treatments have focused on addressing rural populations. A few studies have investigated the effectiveness of videoconferencing in homebound populations, such as disabled adults with dementia or older adults with depression [50,51]. This study suggests that telepsychology methods can also be useful to young employees living in metropolitan areas. Most adults in their 30s and 40s are smartphone savvy, which can allow them to access treatment at their convenience as long as they have a Wi-Fi or LTE (Long-Term Evolution) connection [10]. Videoconferencing via smartphones is also cost-effective as most people already own smartphones and they will be able to save travel costs [52].

To date, a variety of effective stress management programs for employees has been proposed and made available [53]. A recent meta-analysis found that while cognitive behavioral therapy interventions yielded the largest effect sizes, relaxation and meditation techniques were the most popular [54]. A SMART-3RP program that included both of these components has been found to improve resilience and minimize perceived stress in palliative care clinicians, medical interpreters, and resident physicians [44]. The intervention duration for the original SMART-3RP program was 8 weeks; however, this study showed that the intervention was effective only after 4 sessions. Moreover, this intervention was effective and also had a low drop-out rate (videoconferencing condition 14.2%, in-person condition 0% after treatment engagement) compared with previous studies. For comparison, a recent meta-analysis found that the mean completion of workplace psychological treatments was 45%, with a range of 3%-95% [55]. With respect to the 0% drop-out rate in the in-person condition, almost 40% of participants were employees of the hospitals in which the study took place; hence, high accessibility may have contributed to the low drop-out rate.

Although there was no statistical significance at follow-up, the post hoc analysis revealed that the mobile videoconferencing condition had a greater effect on emotional labor than both the in-person and self-care conditions at posttreatment. Emotional labor is a unique type of stress experienced in employees. Brotheridge and Grandey proposed that there may be two sources to job-related stress: emotional demands of the work environment and employees’ ability to control their emotions. This indicates that workers with emotionally demanding jobs and a low capacity for emotional control likely experience the greatest job-related stress [29]. Of the participants who completed the study, 19 (26.4%) were nurses. Given that emotional labor is key to making patients feel safe and comfortable [56], many nurses experience emotional labor, resulting in higher job stress, poorer health, greater self-alienation, and increased frequency of depressive mood [57]. The results of this study suggest that mobile videoconference interventions may be effective in reducing emotional labor in employees, at least in the short-term.

A major concern of videoconference treatment is the quality of therapeutic alliance between patient and therapist [58]. Therapeutic alliance has been defined as collaborative effort by the therapist and patient to facilitate healing [59]. In this study, there were no significant differences in therapeutic alliance ratings between the conditions. Our findings demonstrate that stress intervention via mobile videoconferencing does not compromise therapeutic alliance, which is in line with previous telepsychology research results [60].

There are a few notable limitations to this study. The length of intervention and follow-up interval were relatively short. Thus, this study provides no information on the long-term effect of mobile videoconference interventions. Participants were mostly female and all were Korean, limiting the generalizability of the findings to the male gender and other ethnic groups. We did not exclude participants with depression, insomnia, or anxiety disorders, making the study population clinically heterogeneous; as the sample size was not sufficient for subgroup analyses according to the presence or absence of psychiatric diagnosis, we were unable to evaluate whether psychiatric diagnoses influenced the effect of mobile videoconference treatment. Moreover, we did not measure if the intervention improved any workplace variables, such as work performance, absenteeism, and turnover rate. There is also the possibility of a selection bias caused by the recruitment of highly motivated participants. Lastly, participants and therapists were not blinded to their treatment conditions, which may have caused an expectation bias.

In conclusion, this study demonstrates that videoconferencing- based stress reduction interventions can be effective in employees. Further studies with larger sample sizes and longer follow-up intervals may be helpful for determining the long-term effect of this intervention.

## Appendix

Multimedia Appendix 1 Protocol of stress reduction and resilience enhancement intervention.

Multimedia Appendix 2 Changes in clinical scores across time according to condition.

Multimedia Appendix 3 CONSORT‐EHEALTH checklist (V 1.6.1).

## Abbreviations

AIS: Athens Insomnia Scale
ANOVA: analysis of variance
BRS: Brief Resilience Scale
KELS: Korean Emotional Labor Scale
KOSS: Korean Occupational Stress Scale
MINI: Mini-International Neuropsychiatric Interview
PSS: Perceived Stress Scale
SMART-3RP: Stress Management and Resilience Training: Relaxation Response Resilience Program
