# Supplementary material for: A Mobile Videoconference-Based Intervention on Stress Reduction and Resilience Enhancement in Employees: Randomized Controlled Trial
Source: J Med Internet Res. 2018 Oct 22;20(10):e10760. doi: 10.2196/10760 (PMC6234345; doi:10.2196/10760)
Supplement: Multimedia Appendix 1 [file jmir_v20i10e10760_app1.pdf]

Multimedia Appendix 1. Protocol of the stress reduction and resilience enhancement intervention

Session	Theme	Main contents
1	Understanding stress and relaxation response	- Explanation of the goal of the intervention<br>- Setting rules<br>- Exploring the current stressful situation of the participant<br>- Understanding the body's responses to stress<br>- Understanding the concept of mindfulness and relaxation response<br>- Mindfulness and relaxation exercises<br>- Homework assignment
2	Creating adaptive perspectives through correction of cognitive distortions	- Review of homework<br>- Understanding the concepts of cognitive-behavioral therapy (CBT)<br>- Finding cognitive distortions regarding stress<br>- Correction of cognitive distortions using CBT methods<br>- Mindfulness and relaxation exercises
3	Promoting positivity through a healthy lifestyle	- Review of homework<br>- Healthy eating<br>- Recuperative Sleep<br>- Promoting physical activity<br>- Positive thinking (from pessimism to optimism)<br>- Mindfulness and relaxation exercises
4	Humor, empathy and staying resilient	- Review of homework<br>- Learning about humor as a coping mechanism<br>- Humor Strategies<br>- Tips for staying resilient<br>- Mindfulness and relaxation exercises<br>-Ending comments
