# Supplementary material for: A Mobile Videoconference-Based Intervention on Stress Reduction and Resilience Enhancement in Employees: Randomized Controlled Trial
Source: J Med Internet Res. 2018 Oct 22;20(10):e10760. doi: 10.2196/10760 (PMC6234345; doi:10.2196/10760)
Supplement: Multimedia Appendix 2 [file jmir_v20i10e10760_app2.pdf]

Table 2. Changes in clinical scores across time according to condition.

Measure	Videoconference (n=18), mean (SD)			In-person (n = 27), mean (SD)			Self-care (n=27), Mean (SD)			Condition effect F (*P* value)	Time effect F (*P* value)	Condition × time effect at f/u[a] F (*P* value)	Posttreatment between-condition comparisons		1 mo. f/u between-condition comparisons	
	Pre	Post	1 mo. f/u	Pre	Post	1 mo. f/u	Pre	Post	1 mo. f/u				Conditions	*P* value	Conditions	*P* value
PSS[b]	23.5 (4.2)	17.4 (6.0)	16.4 (3.7)	23.0 (3.3)	15.1 (4.3)	13.9 (4.1)	24.6 (4.1)	20.5 (4.0)	17.4 (4.9)	8.7 (<*.001*)	2.1 (.13)	3.1 (*.03)*	VC[c] vs IP[d]<br>VC vs SC[e]<br>*IP vs SC*	.33<br>.08<br>*.005*	VC vs IP<br>VC vs SC<br>IP vs SC	.15<br>.96<br>.13
KOSS[f]	52.8 (7.7)	46.6 (11.0)	45.3 (10.5)	53.1 (9.4)	44.2 (10.4)	42.5 (9.7)	57.5 (9.3)	53.9 (8.6)	54.1 (11.7)	11.6 (<*.001*)	0.2 (.72)	2.5 (.06)	VC vs IP<br>VC vs SC<br>*IP vs SC*	.39<br>.27<br>*.04*	VC vs IP<br>VC vs SC<br>*IP vs SC*	.34<br>.12<br>*.03*
KELS[g]	61.1 (17.2)	47.5 (17.8)	49.2 (17.1)	57.1 (15.6)	52.0 (12.4)	49.4 (12.6)	59.1 (14.6)	55.2 (19.0)	53.1 (16.6)	0.4 (.66)	0.6 (.54)	2.8 (*.03*)	*VC vs IP*<br>*VC vs SC*<br>IP vs SC	*.03*<br>*.01*<br>.43	VC vs IP<br>VC vs SC<br>IP vs SC	.33<br>.09<br>.28
BRS[h]	16.3 (4.1)	19.6 (5.1)	20.2 (4.4)	16.9 (3.5)	20.2 (3.7)	21.0 (3.3)	17.2 (4.0)	18.3 (3.7)	18.0 (4.3)	2.8 (.07)	2.5 (.09)	3.9 (*.008*)	VC vs IP<br>VC vs SC<br>*IP vs SC*	.92<br>.14<br>*.04*	VC vs IP<br>*VC vs SC*<br>*IP vs SC*	.77<br>*.04*<br>*.001*
AIS[i]	16.3 (3.2)	13.8 (3.1)	14.2 (3.2)	16.7 (4.2)	13.6 (3.2)	12.8 (3.0)	16.6 (4.1)	15.7 (3.6)	15.5 (4.6)	2.6 (.08)	0.1 (.84)	4.5 (*.005*)	VC vs IP<br>VC vs SC<br>*IP vs SC*	.36<br>.08<br>*.03*	VC vs IP<br>VC vs SC<br>*IP vs SC*	.05<br>.11<br>*.009*

[a]f/u: follow-up.
[b]PSS: Perceived Stress Scale.
[c]VC: videoconference condition.
[d]IP: in-person condition.
[e]SC: self-care condition.
[f]KOSS: Korean Occupation Stress Scale.
[g]KELS: Korean Emotional Labor Scale.
[h]BRS: Brief Resilience Scale.
[i]AIS: Athens Insomnia Scale.

Italicized numbers indicate statistical significance (p < .05).
